# 3D Reconstruction and Virtual Reality Is an Acceptable and Feasible Method for Addressing Body Image in Bariatric Metabolic Surgery

**DOI:** 10.1007/s11695-024-07256-8

**Published:** 2024-05-09

**Authors:** Nazrin Assaf, Samantha Scholtz, Ahmed R. Ahmed, Mitchel Krieger, Nasteha Ali, Fernando Bello

**Affiliations:** 1 Department of Surgery and Cancer, Imperial College London, London, UK; 2 Imperial Weight Centre, Imperial College Healthcare NHS Trust, London, UK; 3 Stamford Health, Stamford, USA; 4 West London NHS Trust, Southall, UK

**Keywords:** Bariatrics, Body image, Virtual reality, 3D

## Abstract

**Background:**

Patients living with obesity continue to experience body image dissatisfaction following bariatric metabolic surgery. The underlying reasons are poorly understood but may be due to unmet expectations. Negative body image perception following metabolic surgery leads to poorer psychological and clinical outcomes. This study aims to establish the acceptability and feasibility of three-dimensional (3D) reconstruction and virtual reality (VR) as a method of providing psychological support to bariatric patients to improve body image satisfaction and interventional outcomes.

**Methods:**

Seven participants were recruited from the Imperial Weight Centre. 3D photographs were captured and processed to produce two 3D reconstructed images with 15% and 25% total weight loss. Participants were shown their images using VR and participated in peer group workshops.

**Results:**

Six participants were retained until the end of the study. Five out of six participants agreed the images provided them with a more accurate representation of their body changes and overall appearance following bariatric metabolic surgery. All participants strongly agreed with the group setting and felt VR facilitated discussions on body image. Overall, all participants felt that the use of VR and 3D reconstruction is beneficial in supporting patients to adjust to changes in their body image after bariatric metabolic surgery.

**Conclusions:**

This is the first study to explore and demonstrate that 3D reconstruction and VR is an acceptable and feasible method providing patients with a realistic expectation of how their body will change following significant weight loss, potentially improving body image satisfaction after surgery, as well as psychological and interventional outcomes.

**Graphical Abstract:**

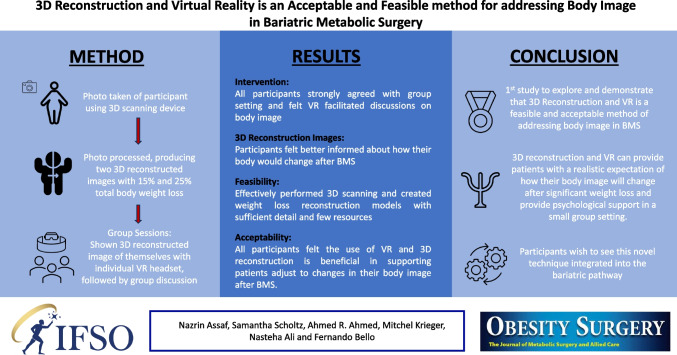

## Introduction

Obesity is a complex multifactorial disease emerging from a struggle between nurture, nature, genetics, and environmental factors [1]. The prevalence of obesity has doubled worldwide since the 1980s across all ages and gender [2]. It is now considered one of the most prominent health threats worldwide [3]. Bariatric metabolic surgery (BMS) has a significant impact on metabolic disease, but also results in significant weight loss, with 30–40% of excess weight lost within the first year, and up to 70% in the 3 years following surgery [4]. For one in five patients living with obesity, body image dissatisfaction is the driving force for surgical intervention. However, recent studies have indicated that BMS patients continue to experience dissatisfaction with their new body and identity due to unrealistic expectations or unmet goals [5, 6]. Negative body image perception plays a central role as a predecessor to obesity through the development of anomalous and unhealthy eating habits [7]. Therefore, negative body image perception and self-stigmatisation post-intervention lead to a recurrence of anomalous eating habits and poor interventional outcomes [7, 8]. BMS plays an important role in treating obesity and its associated complications, but, for it to be successful, the more complex psychological needs such as body image perception need to be addressed.

This pilot study aims to assess the feasibility and acceptability of 3D image reconstruction and virtual reality (VR) as a method of helping bariatric patients manage their expectations and improve body image satisfaction after surgery. This innovative method has the potential to pave the way for developing a platform which may act as a helpful adjunct in bridging the gap between preoperative and postoperative psychological support to improve quality of life, preventing disordered eating, anxiety, and depression after surgery.

## Materials and Methods (Fig. 1)

We aimed to recruit ten patients from the Tier 3 Group (Specialist Weight Management Programme) at Imperial Weight Centre with the aid of a service user (previous patient who has used the service and undergone BMS) from July 2022 to September 2022. Potential participants were screened and sent a participant information sheet. Inclusion criteria were patients aged 18 years and above. Patients who could not provide informed consent, with significant mental instability, or were involved in current or recent research were excluded. Written consent was obtained from all participants (REC 22/LO0422).

**Fig. 1:**
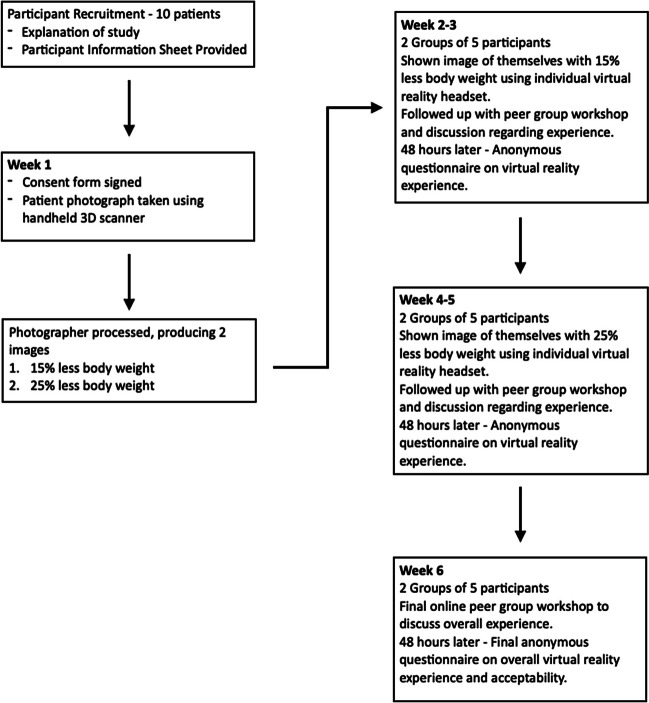
Study method flow chart

3D images of patients in their underwear were captured using a secure password-protected handheld 3D scanning device (iPad Pro 3rd Generation (Apple Inc, CA, USA)). The image was processed to produce two reconstructed images with 15% and 25% less total body weight. The participants were randomised into two groups and engaged in four activities: (i) 1 week after the initial photograph, participants were shown their own image with 15% less body weight using individual VR headsets (Oculus Quest 2 (Meta Platforms Inc, CA, USA)) and participated in a group discussion about their experience; (ii) after 48 h, they completed an anonymous feedback questionnaire (Appendix A) regarding the experience; (iii) each group repeated this process 2 weeks later with their 25% less body weight image. An option was given to compare their original, 15% and 25% less total body weight images; (iv) after 1 week, the participants discussed their overall experience online and provided feedback via an anonymous online questionnaire 48 h later. Fig 1.

### 3D Reconstruction Process

**Fig. 2:**
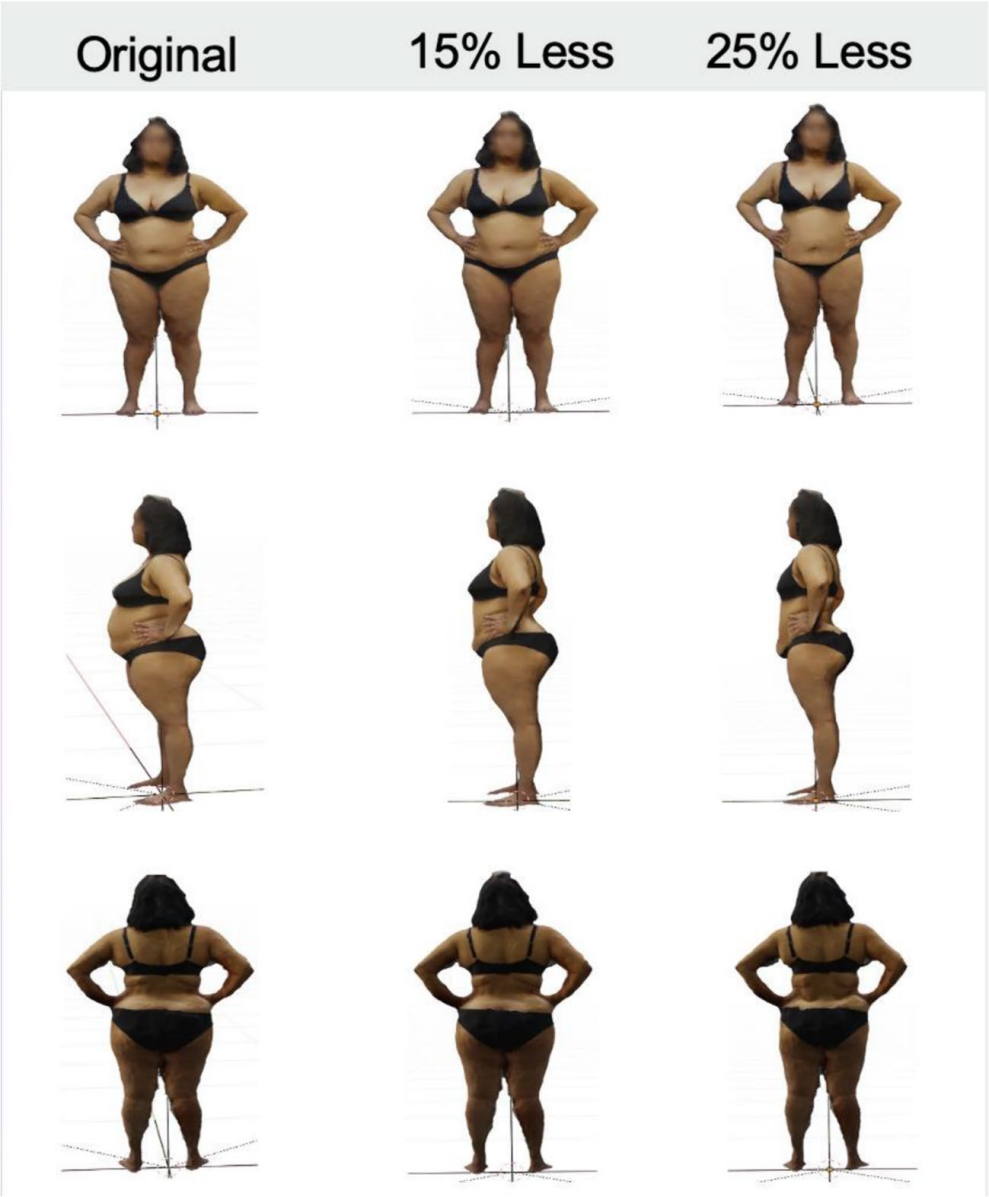
Example of 3D reconstructed models constructed in Blender (v3.0.1)

3D images of the participants were taken on an Apple iPad Pro using the Scaniverse (Version 1.9.3) [9] application. These were exported to Blender (v3.0.1) to crop any excess environment around the images. Fifteen percent and 25% reduction in the original volume was carried out using a combination of automated Python code and manual sculpting depending on the individual’s body shape (Fig. 2). This was based on an understanding of the nature of weight loss, the zones of adherence described by Lockwood [10], and clinical observations from a plastic surgeon specialising in reconstructive surgery following significant weight loss.

### Virtual Reality Environment

**Fig. 3:**
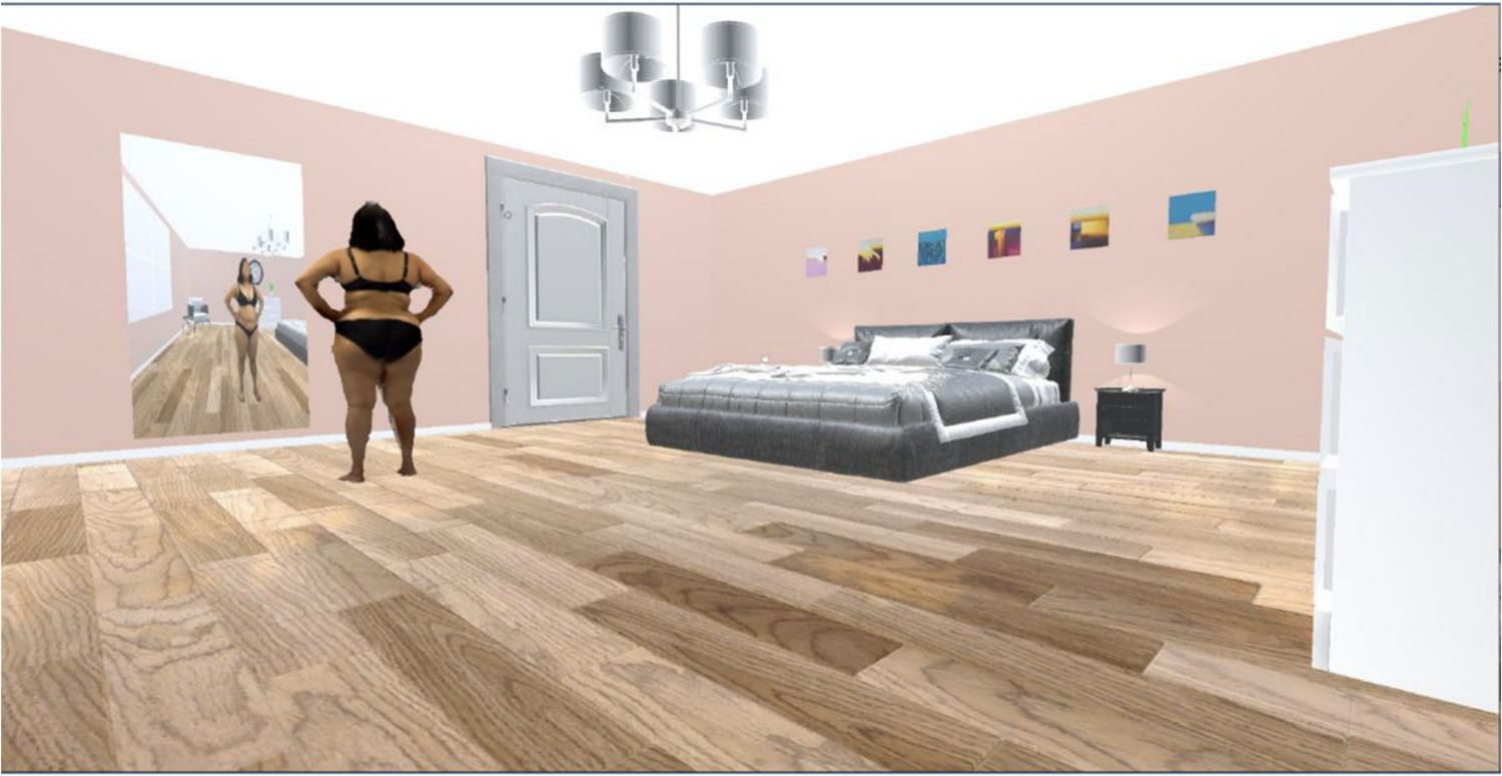
VR scene in Unity (2021.3.0f1)

The VR scene was created in Unity (2021.3.0f1) within a bedroom environment to reflect the most realistic and relatable environment where patients may be semi-clothed. The room was designed with high ceilings and neutral décor to avoid negative connotations. The bed was positioned in the distance keeping in mind the prevalence of sexual abuse in people living with obesity. The window and door in the room avoid a sense of entrapment, and the mirror explores the well-known clinical observation of patients not recognising weight loss changes in their reflection. Each 3D reconstructed model was imported from Blender into the Unity bedroom scene (Fig. 3) and uploaded to the participant’s VR headset, where they were able to navigate the image using a handheld controller.

### Outcome Measure

The primary outcome measure was the acceptability and feasibility of the VR and 3D reconstruction as a method of providing psychological support to bariatric patients. This was based on responses from group discussions and anonymous questionnaires. Qualitative data was analysed using the Framework analysis method.

## Results

### Participant Data

Seven patients were recruited with a median age of 41 (IQR: 13) including six females. One male participant did not attend any further appointments and was excluded from the study. He did not provide a reason for his non-attendance.

### The Study Process

All patients participated in the study to gain motivation, set realistic goals, and manage their expectations of body image after BMS. They all struggled with their body image and felt a visual representation of a realistic weight loss would provide a more positive mindset in the process. All participants agreed with the integration of VR at the beginning of the specialist weight management pathway. Participants reported feeling calm, safe, and respected in the 3D scanning environment in a clinical setting without mirrors. They expressed that, although the study was a big personal commitment with regards to time, it was important to engage and prioritise their own health needs. The biggest barriers were work schedules, being carers, and their own physical health.

#### The Intervention

All participants found the VR headsets enjoyable and easy to use. Two participants reported nausea, but this did not act as a deterrent. Five out of six agreed that the VR visualisation was clear and of good quality. The 3D models provided sufficient detail to recognise the weight loss, but the slight mesh defects were occasionally distracting. All participants reported that the VR bedroom was a realistic and safe space, feeling that a changing room would create anxiety. Interestingly, participants focussed on the 3D models of themselves rather than the mirror reflection, which were harsh and suggestive of a negative relationship with the mirror. One group expressed that it may be beneficial to address these issues within the groups when reviewing their images in real-time. All participants agreed the VR experience helped facilitate key discussions on body image, breaking down initial barriers through all having shared the same experience. Face-to-face sessions were preferable, and this was evident during the last online meeting, where participants no longer felt open or connected with their peers as they were not able to see each other’s bodies, which had brought authenticity to the experience. The recurrent theme was the value of a shared common experience and connecting in real-time with others on the same journey. Loneliness on the bariatric journey was a key feature as some had not shared the decision with their loved ones for fear of judgement and stigma from “taking the easy way out”. The presence of the service user providing a lived experience was invaluable.

#### 15% Total Weight Loss Reconstruction

These images received mixed reviews. One individual felt discouraged and re-focussed their thoughts on a healthier lifestyle rather than weight loss. Another participant did not notice the weight loss but acknowledged in the past they had not noticed their own weight loss. For one participant, it triggered thoughts about the next part of the journey dealing with the resulting skin folds. Only 50% reported that the 15% weight loss reconstructed images provided a better idea of the body changes to expect after surgery, but all participants expressed that overall, they had a more realistic insight. Although participants avoided mirror reflections, five out of six requested to see their original 3D models.

#### 25% Total Weight Loss Reconstruction

These images received positive reviews with five out of six participants agreeing that the images provided a realistic idea and achievable target of their appearance 6–12 months after BMS. Participants accepted that skin folds were a reality, and the images would help them adjust to their potential new body image, having a more positive body image going forward. The images instilled motivation for the journey and encouraged value in their own appearance with the aim of wearing better-fitting clothes and using gym equipment with more confidence.

#### Overall Experience

All participants expressed a positive study experience and strongly agreed they were adequately supported. The group setting created an inclusive environment with safe discussions about body image with shared experiences. The presence of a service user was highly beneficial, allowing discussions with someone who already experienced the journey. All participants expressed that the experience provided a realistic expectation of their appearance following significant weight loss instead of an idealistic one. Rather than this making the participants feel despondent, it was more motivating as it provided a goal which felt realistic and achievable. The majority felt they were better informed of how their body will change after surgery. The weight loss models demonstrated a gradual change, highlighting the bariatric journey as a process not simply an end point. Some participants reported they would be happy with 10–15% weight loss, which is achievable with medication and sufficient to provide health benefits, allowing informed decisions regarding medical versus surgical intervention. Comparisons between their original self, versus 15% and 25% total weight loss models facilitated realistic milestone planning, taking the focus away from the scales. A key realisation was that altering the mindset is even more critical than significant weight loss. Overall, all participants supported the use of VR and 3D reconstruction in helping patients adjust to changes in their body image after BMS.

## Discussion

The key finding of this pilot study suggests that 3D reconstruction and VR can provide patients with realistic expectations of their body image after BMS and psychological support to patients in a small group setting. Recruited participants demonstrated the demand for a novel intervention in the bariatric pathway, feeling strongly that such intervention would be beneficial in helping patients adjust to changes in their body image after surgery, and wishing to see it integrated into the bariatric pathway. From a practical point of view, the study effectively performed 3D scanning and created weight loss reconstruction models with sufficient detail and utilising few resources.

The recruited cohort reflects the population of patients living with obesity who undergo BMS [11]. Service user inclusion has been shown to be lacking in BMS research trials [12], yet it improved the quality of our study.

The primary motivation for participants was the hope of obtaining a realistic visual representation of their appearance after significant weight loss, which was achieved by the study. Previous studies [13–15] have shown the benefits of using visual aids, including VR, to convey health information, improve the patient’s understanding, and achieve better outcomes. This is crucial in the context of body image after BMS. Its complexity remains poorly understood, but we know that body image concerns are more important predictors of well-being postoperatively than objective weight [16]. People living with obesity endure significant stigmatisation and are repeatedly exposed to negative body evaluation shaping their perception of sizes, spaces, and attitudes. Patients may experience “mind–body-lag” after weight loss, but an egocentric and allocentric disconnect makes them continue to feel fat [16]. This was demonstrated in the study when the participants treated their VR avatars with compassion but behaved negatively to its mirror reflection. Most women seeking BMS underestimate their body size, and their postoperative expectations are higher than what can be clinically expected [15, 17]. The presence of skin folds can be a constant reminder that the person used to be obese. After providing a realistic visual representation of their body image following BMS, we can start to dissect their negative connotations about their body, providing tools to allow a disconnect and develop coping strategies following surgical intervention.

This study revealed some unexpected positive findings. There was a risk patients would experience distress on seeing their 3D reconstructed images; however, they were viewed with compassion, and it was clear that patients were kinder to themselves away from the mirror. It broke their idealistic views, which paradoxically allowed them to be more mindful of how to achieve a realistic goal. Furthermore, a less cosmetically pleasing insight made them feel more positive about the journey ahead with a focus on the associated physical health benefits related to metabolic surgery, instead of the cosmetic ones.

## Limitations

The small patient population comprised of females only, reflecting retention of participants in clinical trials, making it impossible to study differences based on gender. There is an inherent bias in the participants who were willing to see realistic changes to their body shape after surgery. This may not be reflective of the bariatric population where many patients harbour psychological struggles. From a technical aspect, the 3D reconstruction was semi-automated requiring manual sculpting, which would not be feasible on a large scale. Further development is required to establish a high throughput system.

## Conclusion

This is the first study to demonstrate that 3D reconstruction and VR are feasible and acceptable methods of addressing body image in BMS. It has been an invaluable experience for all those involved but especially highlighted the importance of co-producing research with a service user. There is a need for a randomised controlled trial with a larger population. This will allow machine learning to accurately predict body image changes after weight loss. A consensus within the bariatric multidisciplinary team is important to consider the timing of integration of this intervention into the bariatric pathway.
